# Towards a Biohybrid Lung: Endothelial Cells Promote Oxygen Transfer through Gas Permeable Membranes

**DOI:** 10.1155/2017/5258196

**Published:** 2017-08-23

**Authors:** Sarah Menzel, Nicole Finocchiaro, Christine Donay, Anja Lena Thiebes, Felix Hesselmann, Jutta Arens, Suzana Djeljadini, Matthias Wessling, Thomas Schmitz-Rode, Stefan Jockenhoevel, Christian Gabriel Cornelissen

**Affiliations:** ^1^Department of Biohybrid & Medical Textiles (BioTex), AME-Helmholtz Institute for Biomedical Engineering, ITA-Institut für Textiltechnik, RWTH Aachen University, Pauwelsstraße 20, 52074 Aachen, Germany; ^2^AMIBM-Maastricht University, Urmonderbaan 22, 6167 RD Geleen, Netherlands; ^3^Department of Medicine, University of Vermont, College of Medicine, Burlington, VT, USA; ^4^Department of Cardiovascular Engineering (CVE), Institute of Applied Medical Engineering, RWTH Aachen University, Pauwelsstraße 20, 52074 Aachen, Germany; ^5^DWI-Leibniz Institute for Interactive Materials, Forckenbeckstraße 50, 52056 Aachen, Germany; ^6^Institute of Applied Medical Engineering (AME), Helmholtz Institute for Biomedical Engineering, RWTH Aachen University, Pauwelsstraße 20, 52074 Aachen, Germany; ^7^Department of Internal Medicine, Section of Pneumology, Medical Faculty, RWTH Aachen University Hospital, Pauwelsstraße 30, 52074 Aachen, Germany

## Abstract

In patients with respiratory failure, extracorporeal lung support can ensure the vital gas exchange via gas permeable membranes but its application is restricted by limited long-term stability and hemocompatibility of the gas permeable membranes, which are in contact with the blood. Endothelial cells lining these membranes promise physiological hemocompatibility and should enable prolonged application. However, the endothelial cells increase the diffusion barrier of the blood-gas interface and thus affect gas transfer. In this study, we evaluated how the endothelial cells affect the gas exchange to optimize performance while maintaining an integral cell layer. Human umbilical vein endothelial cells were seeded on gas permeable cell culture membranes and cultivated in a custom-made bioreactor. Oxygen transfer rates of blank and endothelialized membranes in endothelial culture medium were determined. Cell morphology was assessed by microscopy and immunohistochemistry. Both setups provided oxygenation of the test fluid featuring small standard deviations of the measurements. Throughout the measuring range, the endothelial cells seem to promote gas transfer to a certain extent exceeding the blank membranes gas transfer performance by up to 120%. Although the underlying principles hereof still need to be clarified, the results represent a significant step towards the development of a biohybrid lung.

## 1. Introduction

Chronic respiratory diseases (CRDs) are the third most frequent cause of mortality of noninfectious diseases worldwide, with an increasing incidence and prevalence. CRDs such as chronic obstructive pulmonary disease (COPD), occupational lung diseases, and pulmonary hypertension account for 4 million deaths each year, with COPD representing the major share of over 3 million deaths in 2012. Although various forms of treatment address symptoms and improve the patient's quality of life, CRDs are not curable, progress in time, and may lead to lung failure [1, 2].

In terminal respiratory diseases, lung transplantation is currently the only curative therapy. However, suitable donor organs are limited and transplantation involves risks of graft rejection, infections, and severe side effects of lifelong immune suppression. Hence, lung transplantation is available only to a small group of patients [3, 4].

For patients suffering from respiratory failure, mechanical ventilation is the therapy of choice to ensure adequate gas exchange. Patients who fail mechanical ventilation might be treated with extracorporeal lung support (ECLS) as an alternative treatment. Large blood vessels are accessed and the blood flow is directed through a blood-gas exchanger (oxygenator) enabling the vital gas exchange via a gas permeable membrane [5–9].

Recent advances facilitate short-term use of ECLS systems in acute respiratory distress syndrome (ARDS), COPD exacerbations, as a bridge-to-transplantation, and for the transportation to referral centers. Due to the increasing incidence of chronic lung diseases in general and the lack of suitable donor organs, this concept is also under investigation for long-term lung assist and eventually should allow for destination therapy [5–7, 10].

However, the application is limited to up to 30 days at present time (e.g., 29 days for Novalung™ iLA, Xenios AG, Germany, and 30 days for HLS Set Advanced, Maquet Holding B.V. & Co. KG, Germany) [11, 12]. The complications associated with ECLS comprise inflammatory reactions, hemolysis, hemorrhage, and thrombosis and thus, its application requires continuous intensive care observation. Furthermore, unspecific protein binding to the membrane gradually reduces the efficiency of gas exchange. Both aspects are ascribed to the low hemocompatibility of the gas permeable membranes that are in direct contact with the blood [3, 4, 13–16].

To address this core issue, current research focuses on biohybrid approaches, with endothelialization being at the center of attention [3, 4, 15–20]. The endothelium lines the lumen of blood vessels and is the natural surface known not to elicit inflammatory and coagulatory responses in direct contact with blood. It is composed of a confluent monolayer of endothelial cells (ECs) and regulates the coagulation by tightly controlling the plasmatic coagulation cascade and the adherence of thrombocytes. The intact endothelium also orchestrates the delicate balance between pro- and anti-inflammatory stimuli at its surface [20, 21].

Therefore, lining artificial surfaces with functional ECs (so-called endothelialization) promises physiological hemocompatibility and long-term stability for lung support devices. Previous studies performed both* in vitro *[15, 18, 19, 22, 23] and* in vivo *[24] have already supported the assumption that endothelialization enhances blood compatibility in biohybrid lung devices.

However, the additional cell layer increases the diffusion barrier of the blood-gas interface and thus alters gas transfer. This property is crucial for the design of such a biohybrid device and needs to be elucidated. In order to maximize gas exchange performance, while maintaining an integral EC layer at the same time, we addressed the research question “How does the endothelial cell layer affect the gas exchange?” in this study.

## 2. Materials and Methods

### 2.1. Isolation and Cultivation of HUVECs

In this study, human endothelial cells were derived from umbilical cords (HUVECs) considering ethical commission guidelines of the RWTH Aachen University Hospital after obtaining informed written consent (positive decision of the local ethics committee: EK 019/16).

Isolation of HUVECs was performed under sterile conditions by enzymatic dissociation from umbilical cords. Initially, the umbilical cord was transferred into a sterile buffer solution (100 mM HEPES (Sigma-Aldrich), 140 mM NaCl (Sigma-Aldrich), 2.5 mM KCl, 10 mM glucose (both Merck), and 1 (v/v)% antibiotic-antimycotic solution (ABM, Gibco); pH 7.4; 4°C). Prior to the enzymatic dissociation, the umbilical vein was cannulated from both sides and rinsed with sterile phosphate-buffered saline (PBS, Gibco). For enzymatic dissociation, the vein was filled with 0.2 (w/v)% collagenase in PBS (Sigma-Aldrich) and the cord was incubated for 30 min at 37°C. The dissociated cells were resuspended in endothelial basal medium supplemented with EGM-2 supplements (EBM-2/EGM-2, Lonza) and 1 (v/v)% ABM, seeded into gelatin precoated (2 (w/v)%) cell culture flasks at a cell density of 5 × 10^4^ cells cm^−2^, and finally cultivated in a humidified incubator (5 (v/v)% CO_2_; 37°C). Medium was changed twice a week. Upon 70–80% confluence, HUVECs were subcultivated using 0.25 (w/v)% trypsin/0.02 (w/v)% ethylenediaminetetraacetic acid solution (PAN-Biotech). Eventually, cells in passage 4 were used for this study.

### 2.2. Bioreactor System

**Figure 1:**
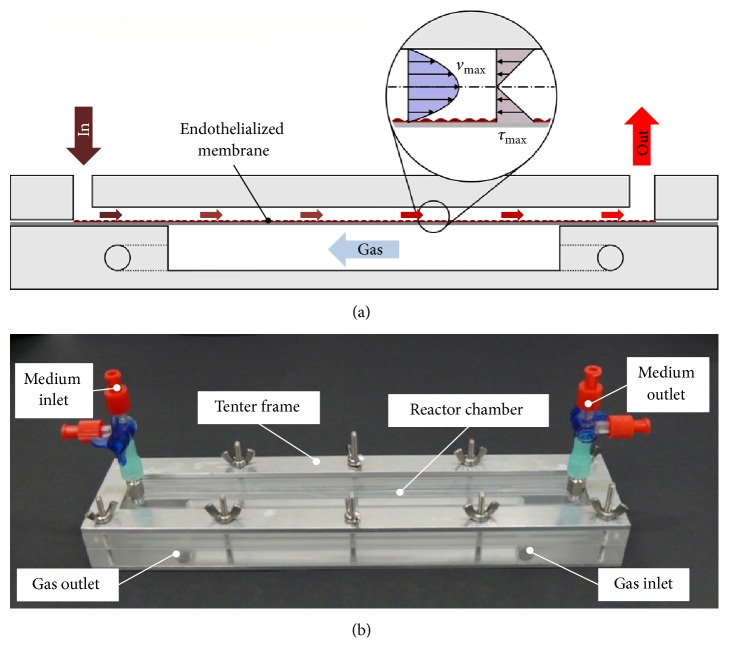
Schematic cross section of the bioreactor system showing the two reactor parts and the endothelialized membrane (a) and the assembled bioreactor (b).

Gas transfer was evaluated in a custom-made modular bioreactor system (depicted in Figure 1) that ensures a laminar flow and a well-defined wall shear stress in the range of physiological values. The reactor itself consists of two chambers, a gas-carrying chamber, and a test fluid-filled chamber, which are separated from one another by a gas permeable membrane. The employed membrane is a nonporous fluorocarbon membrane (lumox®, Sarstedt) with a thickness of 25 *μ*m that allows for cultivation of adherent cells surface treatment (not specified) carried out by the manufacturer.

In terms of a laminar and steady flow of an incompressible frictional fluid between parallel plates, the wall shear stress (WSS) in the test fluid-filled chamber is given by the following [25]:

(1) $$\begin{array}{c} \tau_{0}=\frac{3\cdot \eta \cdot \dot{V}}{2\cdot b\cdot h^{2}}. \end{array}$$

Here, *τ*_0_ is the shear stress at the wall, *η* = 0.693 mPa s is the dynamic viscosity of culture medium at 37°C, $\dot{V}$ is the flow rate, *b* is the width, and *h* is half the distance between the plates. For this chamber, the mean retention time *t*_*t*_ is given by the quotient of the reactor volume *V*_*R*_ to the flow rate $\dot{V}$; hence

(2) $$\begin{array}{c} t_{t}=\frac{V_{R}}{\dot{V}}. \end{array}$$

The parameters for this calculation are given in Table 1.

### 2.3. Static and Dynamic Cultivation in the Bioreactor System

For endothelialization of the membrane, HUVECs (passage 3) suspended in EBM-2/EGM-2 (with 1 (v/v)% ABM) were transferred to the medium chamber of the bioreactor at a concentration of 10^7^ cells mL^−1^. During cultivation (passage 4), the bioreactor was placed in a humidified atmosphere in 5 (v/v)% CO_2_ at 37°C and the gas-carrying chamber was open to this atmosphere. The low chamber volume (and the therein vested low nutrient supply) required an hourly change of medium.

After a two-hour static cultivation, the dynamic cultivation was started by applying a steady and fully developed flow to the cells with hourly increasing flow rates according to Table 2. Their corresponding WSS range from 0.04 to 0.154 Pa (equivalent to 0.4 to 1.54 dyn cm^−2^) and are in the physiological range of human venous vasculature [26].

### 2.4. Gas Transfer Testing

**Figure 2:**
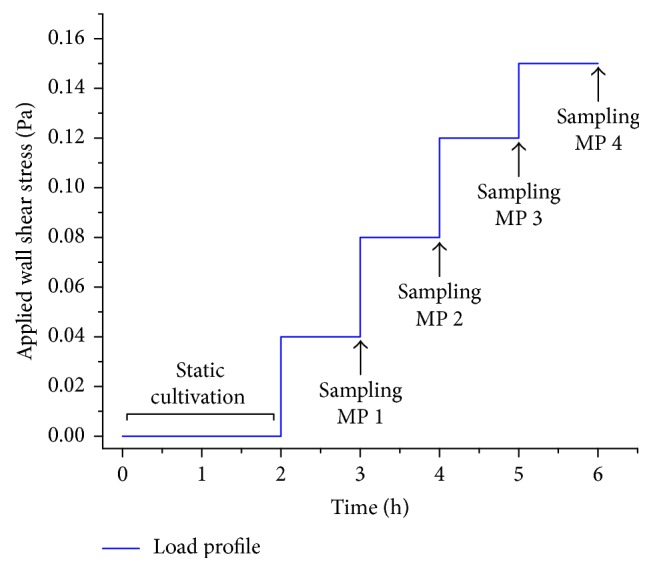
Temporal load profile and sampling times for gas transfer testing at measuring points MP 1 to MP 4.

To evaluate the influence of the EC layer, the oxygenation of the test fluid via the blank membrane was determined first by means of the oxygen transfer rate (OTR). It was followed by an endothelialization of the same membrane, a two-hour static cultivation (as described above), and eventually subsequent testing. In total three independent tests were performed with the temporal load profile of Figure 2.

During testing, the gas-carrying chamber was perfused with pure oxygen (1 bar), while endothelial culture medium (EBM-2/EGM-2 containing 1 (v/v)% ABM) flowed through the other chamber on the principle of counter current. Here, the culture medium met three functions:

1. it provided nutrients to the cells,
2. it sheared them with the flow rate, and
3. it served as test fluid for the gas transfer testing.

Especially for the latter, the culture medium was equilibrated regarding its gas partial pressure to the prevailing incubator conditions (37°C; 5 (v/v)% CO_2_) prior to testing.

Samples of at least 0.5 mL volume were taken in triplicate for every measuring point (MP 1–MP 4) beginning at the outlet (out) and thereupon at the inlet (in) of the reactor chamber. Prior to every sampling, the reactor chamber was perfused with at least three times its volume to ensure equilibrium state. Moreover, the dead volume in the lines was discarded. The samples were sterile filtered and hermetically sealed until analysis in a blood-gas analyzer (ABL 850 Flex, Radiometer) regarding partial oxygen pressure (pO_2_). The maximum time span from sampling to analysis was four hours. Control measurements with a time span of 12 hours between sampling and analysis showed no significant influence on gas analysis (results not shown).

### 2.5. Evaluation of Gas Transfer

According to Henry's law, the oxygen content in culture medium (in contrast to blood) is a function of partial oxygen pressure pO_2_ and the solubility coefficient *α*_O_2__. Thus,

(3) $$\begin{array}{c} O_{2}=\alpha_{O_{2}}{pO}_{2}. \end{array}$$

Substantiated in their similar composition regarding salts and proteins, the solubility coefficient of the respective gas in blood at 37°C was used (*α*_O_2__ = 0.028 mL_O_2__ mL_medium_^−1^ atm^−1^) [27]. Oxygen transfer through the blank (OT_M_) and the endothelialized membrane (OT_M+C_) was computed as the difference of the mean gas content of the inlet and outlet samples:

(4) $$\begin{array}{c} OT=O_{2,out}-O_{2,in}. \end{array}$$

Relating OT to the flow rate $\dot{V}$ of the test fluid yielded the oxygen transfer rate (OTR), with

(5) $$\begin{array}{c} OTR=\dot{V}OT. \end{array}$$

Eventually, to depict the influence of the EC layer to the gas transfer, the difference of both transfer rates was determined:

(6) $$\begin{array}{c} \Delta OTR={OTR}_{M+C}-{OTR}_{M}. \end{array}$$

### 2.6. Bright Field Microscopy

General growth, morphology, and confluence of the EC layer were evaluated with a stereo zoom microscope (Axio Zoom V16, Carl Zeiss) equipped with a CCD camera (AxioCam MRm, Carl Zeiss) and Zen (blue) software. Microscopy was performed after the two-hour static cultivation as well as after every measuring point. This ensured that an integral monolayer has been maintained throughout all experiments.

### 2.7. Immunohistochemistry and Fluorescence Microscopy

Endothelial cells were seeded on lumox membranes and cultivated according to the previously described protocols for static and dynamic cultivation. Samples were fixed in methanol (VWR Chemicals) for 10 min at −20°C and washed thrice with PBS. Nonspecific blocking was conducted by incubation with 3% bovine serum albumin (BSA, Sigma-Aldrich) in PBS for 30 min at room temperature. ECs were stained against CD31 (PECAM-1) and von Willebrand factor (vWf). All antibodies were incubated for 1 h at 37°C. For CD31, the primary antibody (monoclonal, species: mouse, P8590, Sigma-Aldrich, dilution 1 : 100 in 3% BSA PBS) and the secondary antibody (Alexa Fluor 594, goat anti-mouse, A11005, Lifetechnologies, dilution 1 : 400 in 3% BSA PBS) were used. In between steps, samples were rinsed thrice with PBS.

Permeabilization of cell membrane was achieved by three incubations with washing buffer (0.1% Triton, Sigma-Aldrich, in PBS) for 5 min at room temperature.

For vWF, the primary antibody (monoclonal rabbit anti-human, A0082, Dako, dilution 1 : 100 in 3% BSA PBS) and the secondary antibody (Alexa Fluor 488, goat anti-rabbit, A11008, Lifetechnologies, dilution 1 : 400 in PBS) were used. In between steps, samples were rinsed with washing buffer. The cells were counterstained with DAPI (Carl Roth) for 5 min at room temperature and rinsed with PBS. As negative controls, samples were incubated in PBS or washing buffer and the secondary antibody only. Samples were viewed with a fluorescence microscope (AxioObserver Z1, Carl Zeiss) and images were acquired with a high resolution CCD camera (AxioCam MRm, Carl Zeiss).

### 2.8. Statistics

Continuous variables are expressed as mean value ± standard deviation (mean ± SD). For calculated values the Gaussian error propagation was applied. An unpaired two-tailed Student's *t*-test was performed for nonparametric comparison between groups. A *p*-value below 0.05 was considered to be statistically significant (labelled *∗*). Data analysis was performed using commercially available software (Excel 2010, Microsoft).

## 3. Results

### 3.1. Static and Dynamic Cultivation in the Bioreactor System

**Figure 3:**
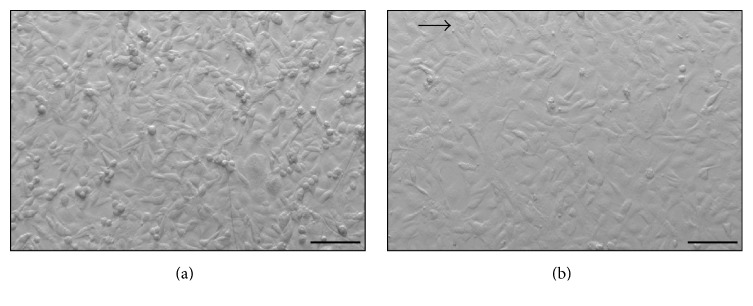
Microscopic images of EC layer. (a) shows confluent polygonal cobblestone-like HUVECs after 2 h of static cultivation. (b) shows an aligned and stretched cell morphology after gas transfer testing with a maximal WSS of 0.15 Pa. Arrow indicates direction of flow (scale bar: 100 *μ*m).

Figure 3 shows the EC layer after two hours of static cultivation (a) and after gas transfer testing with a maximal wall shear rate of 0.15 Pa. In both cases the cell layer is confluent. Thus, the monolayer withstood the applied loads and allowed for gas transfer tests. Shear stress induced the typical modification from a polygonal, cobblestone-like morphology to aligned and flattened cells for all experiments.

**Figure 4:**
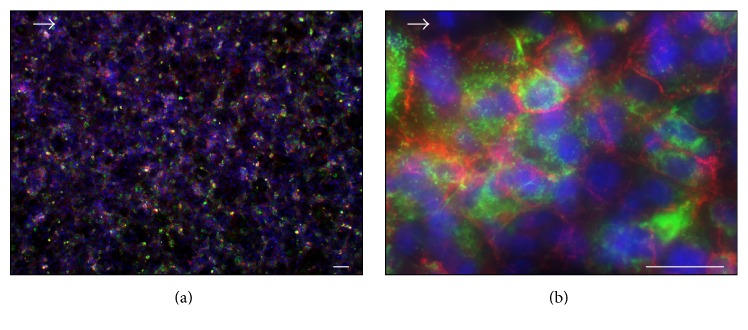
Immunohistochemical staining of endothelial cell layer against CD31 (red) and vWF (green). Cell nuclei were stained with DAPI (blue). Arrow indicates direction of flow. Scale bars: 10 *μ*m (a) and 5 *μ*m (b), respectively.

Figure 4 shows the immunohistochemistry staining of the EC layer after four hours of dynamic cultivation with a maximum wall shear stress of 0.15 Pa. The cell layer stained positive against typical EC markers CD31 and vWF verifying the endothelial cell phenotype. Due to the short cultivation period, the staining intensity of the cell layer varied and was diminished in general.

### 3.2. Evaluation of Gas Transfer

The gas analysis data from the gas transfer tests is given in Supplementary Table S1 (see Supplementary Material available online at https://doi.org/10.1155/2017/5258196) for both experimental setups (index M for blank membrane, index M + C for endothelialized membrane) in terms of mean pO_2_ of the three experiments each prior and after oxygenation, that is, at the inlet and outlet, respectively.

**Figure 5:**
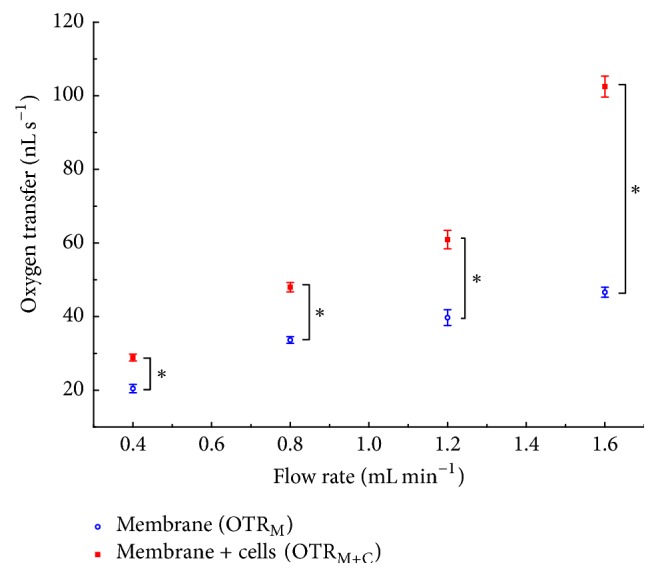
Mean oxygen transfer rates for endothelialized (OTR_M+C_) and blank (OTR_M_) membranes. Error bars indicate standard deviation. Asterisk indicates *p* < 0.05.

The computed oxygen transfer rates (OTRs) of the blank and the endothelialized membrane as well as their relation are given in Supplementary Table S2 and Figure 5. With respect to the OTR, both experimental setups exhibited positive transfer rates that increased continuously with increasing flow rate. The latter is based on the dependence of the OTR on the flow rate (cf. $OTR=\dot{V}OT$) and holds for OTR_M_, as well as OTR_M+C_. The overall maximal OTR was 102.5 ± 2.9 nL s^−1^ for M + C_1_.

Moreover, the direct comparison with the blank membrane yielded in absolute terms greater transfer rates for the endothelialized membrane. It is evident that the performance of the endothelialized membrane exceeded the gas transfer of the blank membrane by up to 120% for MP 4. Furthermore, a disproportionate increase of OTR_M+C_ compared to OTR_M_ with increasing flow rate was ascertainable. This increase in OTR only held true for the endothelialized membrane and was therefore independent of the flow rate.

## 4. Discussion

With regard to the development of a biohybrid lung, the influence of an EC layer on gas exchange performance of a gas permeable membrane was evaluated. For this purpose, subsequent testing of blank and endothelialized membranes in a bioreactor with endothelial culture medium as a test fluid was performed.

Effective gas transfer testing requires an integral monolayer for high reproducibility and accurate determination of a cell layer's impact. The seeded ECs developed a confluent and flow-resistant layer before and after gas transfer measurements as proven by bright field and fluorescence microscopy.

The gas transfer tests show the suitability of the bioreactor to oxygenate the test fluid in the measuring range for both experimental setups. The absolute values of the gas transfer are small due to the dimensions of the bioreactor and especially due to the selected gas permeable membrane. As a fluorocarbon bulk material, the lumox film features relatively poor gas permeability that is comparable to other fluorocarbon materials such as polytetrafluoroethylene (PTFE; oxygen permeability coefficient (OPC) 178 cm^3^ mm m^−2^ day^−1^ atm^−1^). These materials are opposed to silicones, which have very good gas permeabilities (OPC 19.685 cm^3^ mm m^−2^ day^−1^ atm^−1^) and are therefore widely used in gas exchange applications [28]. Prospectively, a change in membrane material will increase the overall gas exchange. Nevertheless, the obtained results show only small standard deviations and enable an initial evaluation of the bioreactor system as a model system for a biohybrid lung assist device.

In addition, it has to be considered that all experiments have been carried out with culture medium and that its rheological properties as well as its gas binding and gas transport characteristics differ from blood. While oxygen can merely be physically dissolved in culture medium, it is mainly chemically bound to the hemoglobin in blood that consequently possesses a much bigger oxygen transport and binding capacity. This limits the transferability of the test execution to later application as a biohybrid lung. However, this approach has been chosen as a necessary first experimental step because medium trials—unlike blood—allow for long-term trials and microscopic control of cell layer integrity. Hence, it allows for investigation of fundamental gas transfer characteristics via an endothelialized gas permeable membrane.

Contrary to expectations as well as observations from various groups, who state that the cell layer has no or solely decreasing effect on gas transfer ([3, 15, 18, 22] (all in blood); [16] (in PBS)), the performance of the endothelialized membrane increased significantly compared to the blank membrane.

The endothelialized membrane yields (i) in absolute terms larger and (ii) relatively stronger increasing gas transfer rates compared to the blank membrane. The underlying principles of both effects still need to be clarified.

The absolutely larger values (i) may be due to induced secondary flows in the vicinity of the wall that increase mass transfer in general. This effect is already utilized in modern oxygenators to disrupt the blood side concentration in the boundary layer [29]. It has to be assumed that the positive influence of the EC on the mass transfer in the proximity of the membrane is predominant to any other influencing factors concerning the increased diffusion barrier of the oxygen through the membrane-cell-construct.

The second observation (ii) highlights the fact that the influence of the endothelialization on the gas transfer performance is not constant but a function of flow volume. This might be due to a shear stress induced modification of cell morphology as can be seen in Figure 3. Higher flows correspond to stronger mechanical stimuli promoting the alignment of the cells. Hence, the cell height and therefore the diffusion path are reduced. Barbee et al. already showed that a shear stress of 1.2 Pa reduces the cell height of EC by about 50% and that the cobblestone-like morphology changes to an aligned shape with an overall furrowed surface of the monolayer [30, 31].

With regard to the predominant assumption that the endothelialization merely increases the hemocompatibility but decreases the gas transfer performance due to an increased diffusion barrier, the presented results are of great relevance to the development of biohybrid lung assist devices.

In order to make use of this prevailing effect, the flow and mass transfer conditions in the vicinity of the gas permeable membrane have to be adapted to prospective constructions of biohybrid lungs. However, the transfer to blood trials as well as the appropriate use of this gas exchange promoting effect requires a basic understanding of the underlying mechanisms. Thus, the flow conditions as well as the mass transfer in the vicinity of the membrane need to be further investigated.

## 5. Conclusion

This study presents a modular bioreactor system suitable for the evaluation of gas transfer performance of endothelialized membranes. In a first experimental step, a confluent and flow-resistant EC layer has been established and the oxygen transfer rates of blank and endothelialized cell culture membranes have been determined.

Contrary to expectations, the performance of the endothelialized membrane exceeds the gas transfer of the blank membrane by up to 120%. Hence, the monolayer shows a gas transfer promoting effect that is even increasing with flow rate.

The underlying principles or mechanisms are not fully understood yet and need to be addressed in further experiments. In particular, it has to be investigated whether this effect also occurs with oxygenator membranes, which have significantly higher gas permeability. Still, the results form the basis for further experiments and present a significant step towards the development of a biohybrid lung.

## Supplementary Material

Table S1: Mean oxygen partial pressure for blank and endothelialized membranes.
Table S2: Oxygen transfer rates of blank and endothelialized membranes.

## Figures and Tables

**Table 1:** Parameters of the bioreactor concerning flow calculations.

Parameter	Unit	Bioreactor
Height *H* = 2 h	mm	0.3
Width *b*	mm	8
Length *L*	mm	225
Area *A*	cm^2^	19.9
Volume *V*	mL	0.60

**Table 2:** Measuring points as well as their characteristic parameters for gas transfer tests.

Measuring point MP	Flow rate $\dot{V}$ (mL min^−1^)	Residence time *t*_*t*_ (s)	Wall shear stress *τ*_0_ (Pa)	Time of sampling (h)
1	0.4	90	0.04	3
2	0.8	45	0.08	4
3	1.2	30	0.12	5
4	1.6	22	0.15	6
