# Supplementary material for: Towards a Biohybrid Lung: Endothelial Cells Promote Oxygen Transfer through Gas Permeable Membranes
Source: Biomed Res Int. 2017 Aug 23;2017:5258196. doi: 10.1155/2017/5258196 (PMC5587952; doi:10.1155/2017/5258196)
Supplement: Supplementary file 1 — Table S1: Mean oxygen partial pressure for blank and endothelialized membranes. Table S2: Oxygen transfer rates of blank and endothelialized membranes. [file 5258196.f1.pdf]

Towards a biohybrid lung: Endothelial cells promote oxygen transfer through gas permeable membranes

Supplementary Data

Table S1: Mean pO_2 of three experiments (index 1, 2, or 3) for both experimental setups, i.e. for the blank (index M) and the endothelialized (index M+C) membrane, in dependence of flow rate and location of sampling (inlet or outlet)

**Flow rate (mL min^{-1})**	**Sampling location**	**$pO_{2, M}$ (mmHg)**			**$pO_{2, M+C}$ (mmHg)**		
		**M_1**	**M_2**	**M_3**	**$M+C_1$**	**$M+C_2$**	**$M+C_3$**
0.4	In	169.00 ±1.00	172.00 ±1.00	186.00 ±1.73	179.33 ±0.94	165.33 ±0.94	182.00 ±4.97
	Out	293.00 ±10.58	244.67 ±7.64	239.00 ±3.00	329.33 ±9.84	289.67 ±1.70	260.33 ±2.05
0.8	In	168.67 ±1.25	170.33 ±0.47	185.67 ±2.36	175.33 ±1.70	178.67 ±2.87	181.67 ±0.47
	Out	278.00 ±3.27	224.33 ±2.05	227.67 ±2.87	299.33 ±2.87	289.67 ±4.11	239.67 ±4.78
1.2	In	166.00 ±1.41	169.67 ±0.47	183.00 ±2.16	176.33 ±1.25	166.00 ±1.41	183.33 ±2.62
	Out	254.67 ±1.70	209.67 ±7.41	216.00 ±3.56	282.33 ±6.80	255.67 ±3.09	235.67 ±6.24
1.6	In	166.00 ±1.63	168.67 ±0.47	183.33 ±0.47	176.33 ±0.47	162.33 ±0.47	182.33 ±0.47
	Out	237.00 ±1.41	209.67 ±1.70	213.67 ±3.09	280.67 ±2.87	247.67 ±3.40	232.67 ±0.47

*Table S2: Oxygen transfer rates (OTRs) of the blank and the endothelialized membrane as well as the absolute difference of both OTRs, all OTR_{M+C} are significant to OTR_M ($p < 0.05$, labelled *) throughout the measuring range*

**Flow rate (mL min^{-1})**	**OTR_M (nL s^{-1})**	**OTR_{M+C} (nL s^{-1})**	**ΔOTR (nL s^{-1})**
0.4	20.4±1.1	28.9±0.9*	8.4±1.5
0.8	33.6±0.9	48.0±1.3*	14.4±1.6
1.2	39.7±2.2	60.9±2.5*	21.2±3.3
1.6	46.6±1.4	102.5±2.9*	55.9±3.2
